# Extracellular Vesicles as Nanotherapeutics for Parkinson’s Disease

**DOI:** 10.3390/biom10091327

**Published:** 2020-09-16

**Authors:** Loredana Leggio, Greta Paternò, Silvia Vivarelli, Francesca L’Episcopo, Cataldo Tirolo, Gabriele Raciti, Fabrizio Pappalardo, Carmela Giachino, Salvatore Caniglia, Maria Francesca Serapide, Bianca Marchetti, Nunzio Iraci

**Affiliations:** 1 Department of Biomedical and Biotechnological Sciences (BIOMETEC), University of Catania, Torre Biologica, Via Santa Sofia 97, 95125 Catania, Italy; loredanaleggio@unict.it (L.L.); greta.paterno.gp@gmail.com (G.P.); silvia.vivarelli7@gmail.com (S.V.); gabr_rac92@hotmail.it (G.R.); fabriziopappalardo@hotmail.it (F.P.); serapide@unict.it (M.F.S.); 2 Neuropharmacology Section, OASI Research Institute-IRCCS, 94018 Troina, Italy; flepiscopo@oasi.en.it (F.L.); ctirolo@oasi.en.it (C.T.); carmelagiachino@libero.it (C.G.); scaniglia@oasi.en.it (S.C.)

**Keywords:** Parkinson’s disease, neurodegeneration, extracellular vesicles, exosomes, biomarkers, nanotherapeutics, nanodrugs, nanoparticle, cell-free therapy

## Abstract

Extracellular vesicles (EVs) are naturally occurring membranous structures secreted by normal and diseased cells, and carrying a wide range of bioactive molecules. In the central nervous system (CNS), EVs are important in both homeostasis and pathology. Through receptor–ligand interactions, direct fusion, or endocytosis, EVs interact with their target cells. Accumulating evidence indicates that EVs play crucial roles in the pathogenesis of many neurodegenerative disorders (NDs), including Parkinson′s disease (PD). PD is the second most common ND, characterized by the progressive loss of dopaminergic (DAergic) neurons within the Substantia Nigra pars compacta (SNpc). In PD, EVs are secreted by both neurons and glial cells, with either beneficial or detrimental effects, via a complex program of cell-to-cell communication. The functions of EVs in PD range from their etiopathogenetic relevance to their use as diagnostic tools and innovative carriers of therapeutics. Because they can cross the blood–brain barrier, EVs can be engineered to deliver bioactive molecules (e.g., small interfering RNAs, catalase) within the CNS. This review summarizes the latest findings regarding the role played by EVs in PD etiology, diagnosis, prognosis, and therapy, with a particular focus on their use as novel PD nanotherapeutics.

## 1. Introduction

Parkinson’s disease (PD) is the second most common chronic neurodegenerative disorder (ND) after Alzheimer’s [1]. PD affects about 1–2% of the population over the age of 60 years [2]. Globally, PD incidence is increasing because the life expectancy is longer than in the past, which results in a larger elderly population [3,4]. In fact, according to the World Health Organization, the total of individuals over 65 years will double between 2000 and 2050 [5]. In the near future, PD will affect not only people in the Western industrialized countries, but also those living in the developing countries. For this reason, the number of people with PD is estimated to exceed the 10 million count by 2030 [3,4,6]. The causes and mechanisms involved in PD onset and progression are ill-defined, and currently there is no cure available to stop or reverse PD progression [7].

The clinical symptoms of PD were characterized for the first time in 1817 by the British doctor James Parkinson, who described this condition as a “shaking palsy” [8]. Fifty-five years later, in 1872, the neurologist Jean Martin Charcot acknowledged the important studies conducted by Parkinson, thereby renaming the disease after him [9]. Generally, people affected by PD display the “classical” motor symptoms, i.e., resting tremor, rigidity, bradykinesia, and postural instability. These symptoms are the main result of the progressive degeneration of dopaminergic (DAergic) neuronal cell bodies in the Substantia Nigra pars compacta (SNpc) within the midbrain, and their terminals in the striatum. As a consequence, the neurodegeneration leads to a chronic dopamine (DA) neurotransmitter depletion [10]. As for other NDs, PD is characterized by the accumulation of misfolded protein aggregates, primarily α-synuclein (α-Syn), which forms inclusions (called Lewy bodies) and dystrophic neurites (called Lewy neurites), localized within both nigral and extranigral neurons [11]. These agglomerates contribute to the development of the motor dysfunctions [12], whose severity is correlated with the different PD phenotypes [13,14]. The presence of abnormal α-Syn aggregates in other brain regions may explain the appearance of non-motor symptoms, some of which occur several years before the classical clinical features of PD [15]. Gastrointestinal issues, depression, psychosis, sleep disorder and cognitive impairment are among the non-motor PD symptoms which may negatively affect overall quality of life [16]. Importantly, the different range and onset of the non-motor symptoms during PD progression reflect distinct PD phenotypes [14]. In this regard, in 2015 “The International Parkinson and Movement Disorder Society” defined several criteria to identify and stratify PD, which were validated by many clinical studies [17,18,19].

A chief hallmark of PD is neuroinflammation. Glial cells, particularly activated astrocytes and reactive macrophages/microglial cells, are key players during both the onset and the progression of the disease [20,21,22,23]. Remarkably, depending on the nature of the signal molecules released in the microenvironment, glial cells may acquire either a “beneficial” or a “destructive” phenotype, with critical consequences for DAergic neuronal vulnerability against several harmful stimuli [20,21,22,23,24,25,26].

These cell-to-cell interactions within the brain may be mediated by different strategies of intercellular signaling. The release of soluble factors in the extracellular milieu is one of the most studied mechanisms used by cells to exchange information. More recently, the secretion of extracellular vesicles (EVs) emerged as an additional pivotal strategy to deliver complex molecular messages between cells. EVs are membranous nanoparticles released in the extracellular space by almost all types of cells, deriving from animals or plants, as well as produced by several microorganisms [27,28]. The exact definition of EVs evolved during the last three decades. In fact, a wide variety of subtypes of vesicles have been discovered. Moreover, many technical advancements over the years improved their characterization at the molecular level [29]. EVs have been demonstrated to play several roles in cellular biology. On one hand, EVs might work as “waste system”, but on the other hand they represent carriers delivering the “molecular information” within the microenvironment [30]. This particular aspect attracted the attention of researchers, with the aim to understand how EVs are involved in the cell-to-cell communication, and eventually to explore their potential as cargoes for therapeutic purposes.

The discovery of EVs dates back to more than 50 years ago when Chargaff and West, in 1946, observed the presence of procoagulant platelet-derived particles [31], called later “platelet dust” by Wolf in 1967 [32]. In 1983, Harding and colleagues demonstrated the existence inside rat reticulocytes of multivesicular endosomes that fused back with the plasma membrane to release their vesicular content [33]. These particular vesicles were also obtained in the same year by Pan and Johnson after 1h of 100,000× *g* ultracentrifugation, starting from sheep reticulocytes-derived supernatants [34]. Historically, larger attention has been posed on exosomes, small vesicles with a size ranging between 30–150 nm. Exosomes are produced by the inward budding of the endosomal compartment, thus forming the so-called “multivesicular body” (MVB). These intraluminal vesicles are subsequently released outside the cell after the fusion of the MVB with the plasma membrane. These released exosomes, which correspond to the vesicles observed by Harding in 1983 [33], once outside the producer cell, can be engulfed by adjacent, as well as by distant target cells [35].

As outlined, other types of vesicles have been characterized, such as microvesicles, apoptotic bodies, ectosomes and others [36]. These vesicles differ from the exosomes in their biogenesis, being released directly from the plasma membrane with a shedding mechanism. These shedding vesicles have a dimension between 50 and 1000 nm that partially overlaps with the exosome size. For this reason, to date, it is not possible to separate pure subtypes of EVs [37].

**Figure 1:**
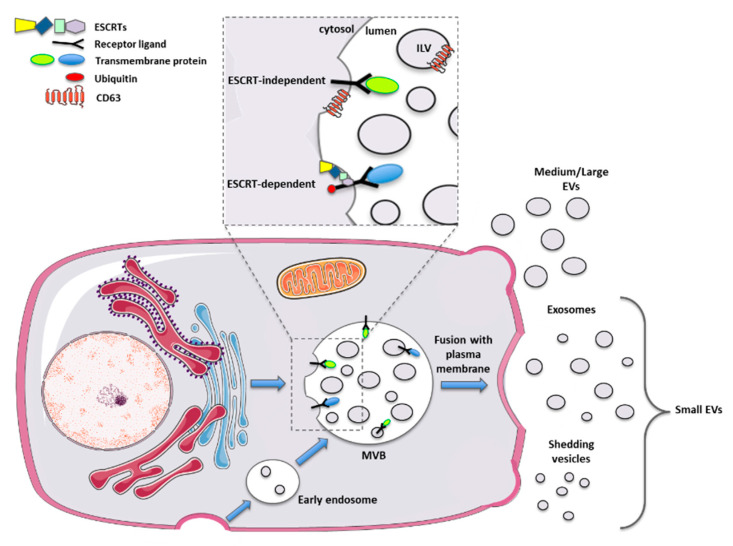
Schematic representation of biogenesis and release mechanisms for different types of extracellular vesicles.

This issue may be solved by the identification of specific EV surface markers. Initially, tetraspanins such as CD63 and CD81 were recognized as specific markers for exosome biogenesis. Further studies found these proteins expressed by additional categories of vesicles, making the identification of specific subtypes not yet feasible [38,39]. Therefore, the literature in the last 15 years reflects all the issues mentioned above. The latest guidance paper, released in 2018 from the International Society for Extracellular Vesicles, defined new important advices for scientists working with EVs [40]. In particular, a new nomenclature has been proposed, based merely on the size of the EVs, thus dividing them into small vesicles (s-EVs < 200 nm) and medium and large vesicles (m/l-EVs > 200 nm) (Figure 1).

Another important aspect deeply investigated in the EV field concerns the analysis of their content. The cargoes delivered via EVs reflect the current “status” of the donor cell and can change in response to specific modifications in the microenvironment. In particular, EVs contain nucleic acids, both DNA (chromosomal or mitochondrial) [41,42] and RNAs (mRNAs, small ncRNAs, such as microRNAs etc.), potentially able to regulate the gene expression of the target cells [43,44]. Moreover, these vesicles transport proteins, metabolites, and lipids, whose identity changes in response to several stimuli [45]. However, the role played by EVs in both physio- and pathological conditions is still debated, since they can be either beneficial or detrimental, depending on the specific context in which they are investigated [46].

In NDs, EVs were initially characterized as vehicles for misfolded or dysfunctional mutant proteins, such as amyloid-beta oligomers in Alzheimer’s disease (AD) [47], SOD1 in amyotrophic lateral sclerosis (ALS) [48], or α-Syn in PD [49]. In line with the dual role described for glial cells, this view was gradually challenged in the last decade by the demonstration that EVs can play relevant neuroprotective functions in several degenerative conditions (including PD), as we and others recently reviewed [50,51].

In this review, the latest findings regarding the roles played by EVs in the development of PD, and their possible use as novel PD biomarkers are provided. In particular, we will focus on the therapeutic potential of both natural and engineered EVs, and the possibility to use them as advanced drug delivery systems in PD.

## 2. PD Risk Factors are Linked to EV Biology

Currently, the causes responsible for the progressive degeneration of midbrain DAergic neurons are poorly understood. Aging, inflammation, genetics, and environmental toxicity are prominent etiological factors in PD development. In particular, aging represents the most critical vulnerability factor for PD, whereby the age-dependent dysregulation of critical cellular functions within the midbrain microenvironment (e.g., inflammation, oxidative and nitrosative stress, proteasome/lysosome dysfunctions) significantly contributes to the progressive neuronal deterioration observed in PD [52,53,54].

Notably, emerging studies on aging mouse models of PD, based on the exposure to the environmental neurotoxins 1-methyl-4-phenyl-1,2,3,6-tetrahydropyridine (MPTP) or 6-hydroxydopamine (6-OHDA), clearly indicate that aged glial cells lose their neuroprotective, pro-neurogenic, and regenerative functions, thereby contributing to the inflammatory and degenerative processes during PD onset and progression [21,23,55,56,57,58,59,60]. In fact, both reactive astrocytes and microglial cells play key roles during PD nigrostriatal degeneration/self-repair. These glial cells are located within the striatal subventricular zone (SVZ), a major neurogenic niche of the adult brain, and the peri-aqueductal ventral midbrain (VMB) region, enriched in DAergic neuroprecursor cells [21,24,26,52,57,58,59,60].

Significantly, in the context of SNpc, reactive astrocytes and microglia represent the key “cellular-hub” able to handle crucial cellular functions—including oxidative and endoplasmic reticulum stress, mitochondrial, lysosomal, proteasomal, and autophagic activities—all converging to α-Syn aggregation and spreading [11,22,26,57,61,62,63,64]. Astrocytes are pivotal cells in maintaining the homeostasis of the microenvironment in the central nervous system (CNS). They provide physical and metabolic support to neurons by helping them to accomplish their complex functions. Moreover, astrocyte functions are selectively linked to the brain region, the type of brain injury, as well as to the age and the sex of the individual [65,66,67,68]. Importantly, they mediate neuroprotective effects via the release of growth and neurotropic factors, antioxidants and anti-inflammatory molecules [20,22,24,26,65,69,70]. For instance, astrocytes from the ventral midbrain are pivotal for the development of DAergic neurons, thanks to the release of an array of pro-survival and neuroprotective molecules [71,72,73,74,75], such as the glial-derived neurotrophic factor (GDNF) [76] and the basic fibroblast growth factor (bFGF) [77], whose levels are altered in PD patients [78,79]. Remarkably, both astrocyte-derived wingless-type mouse mammary tumor virus (MMTV) integration site 1 (Wnt1) and Wnt1/ β-catenin signaling network contribute to astrocyte-neuron interactions in PD. In this context, the Wnt pathway represents a vital cascade, in turn able to promote DAergic neurogenesis, DAergic neuron survival and immunomodulation via a bidirectional glia-neuron cross-talk [21,23,24,25,26,52,55,56,57,58,59,60].

Following brain injury, astrocytes undergo gene expression changes resulting in an activated phenotype and a “reactive astrogliosis” [80]. Almost two decades ago, the concept of a dual beneficial/harmful role of reactive astrocytes and microglia in NDs, including PD [20], paved the way to further characterization of the phenotype-dependent ability of glial cells to deliver either neuroprotective or detrimental molecules for neuronal health and survival [20,22,24,51,81]. As for microglial cells [82], Liddelow and colleagues in 2017 described two different kind of reactive astrocytes, the A1 harmful phenotype and the A2 protective one [83]. Accordingly, A1 astrocytes are involved in many NDs, particularly PD, and display detrimental and neurotoxic functions induced in turn by activated microglia [83]. Microglia—the resident macrophage-like immune cells of the CNS—monitor the cerebral microenvironment through a constant interaction with astrocytes, neurons, and blood vessels, contributing to maintain the homeostatic balance in the brain [22,26,84,85,86].

With age and under neurotoxic stimuli, the microglial cells—similarly to the polarization described for astrocytes—shift to the harmful M1 phenotype and release pro-inflammatory cytokines such as tumor necrosis factor α (TNF-α), interleukin 1β (IL-1β) and IL-6 [22,26,87]. The simultaneous production of reactive oxygen species (ROS) results in a higher oxidative stress and inflamed microenvironment for surrounding neurons [20,22,26,56,57,88,89]. Notably, microglia shows the highest density in the SNpc, suggesting a likely harmful milieu that may predispose DAergic neurons to neuroinflammation-dependent degeneration [22,26,53,57,75,82,90,91]. On the contrary, the M2 polarized microglia are associated with the release of anti-inflammatory cytokines (e.g., IL-4 and IL-10), neurotrophic factors (e.g., BDNF and IGF-1) and extracellular matrix proteins (e.g., fibronectin) [55,57,92]. As a corollary, both activated and aged glial cells secrete EVs capable of exerting both pro- and anti-inflammatory mechanisms to balance immune reactivity in the brain [51,93]. Hence, glial-derived EVs powerfully regulate the inflammatory microenvironment thanks to a panel of immunomodulatory cargoes, which may further direct the glial status to an A1/M1 or A2/M2 phenotype, with crucial consequences for neuronal survival and health, as recently reviewed in [51].

Beside aging, a substantial proportion of PD cases is driven by genetics. Indeed, about 10–15% of patients have a familial history of PD, which indicates the possibility of inheriting such risk factors. On the contrary, the majority of PD cases are considered idiopathic, and likely result from a complex array of interactions between dysfunctional genes and a growing list of environmental risk factors [22,23,26,57,94,95]. In both the familial and idiopathic forms of PD, common molecular pathway alterations have been found. As mentioned before, dysfunctions in the lysosomal and/or ubiquitin-proteasome system, increased levels of oxidative stress, impairments in the mitochondrial respiratory mechanisms and synaptic vesicle-recycling pathway, represent only a few examples of the altered functions existing in both idiopathic and familial PD. As a matter of fact, a complex panel of gene-environment interactions heavily contribute to PD onset and/or progression [22,26,57,96]. For example, living in rural areas and farming may increase the chances to be exposed to pesticides. Some examples are: Rotenone, which leads to mitochondrial complex I dysfunction, and Paraquat, which induce ROS formation. Both environmental exposures represent risk factors for PD [97,98,99].

### 2.1. Genetic Susceptibility in PD

As anticipated, genetics plays an important role in the etiology of PD [100]. Many genes are linked to PD pathogenesis, including an increasing number of novel genetic mutations found associated with specific geographic areas [101].

The first and most characterized PD-linked gene is the *SNCA* gene (*PARK1*), encoding for α-Syn [102,103,104]. Toxic aggregates of this protein are found in PD brains, heavily involved in the insurgence of neuroinflammation and, ultimately, in the clinical symptoms of PD. As expected, several mutations for this gene have been found in PD patients, some of them in familial early onset PD (EOPD), including the missense mutation A53T [105,106]. In particular, the A53T mutation is the most prevalent one, showing faster aggregation kinetics than wild type α-Syn [107].

*PARK2* or Parkin is another gene whose mutations are found in approximately 50% of familial cases with EOPD and ~15% of sporadic EOPD [108,109,110]. *PARK2* encodes for the Parkin RBR E3 Ubiquitin Protein Ligase which is involved in the proteasomal degradation pathway [111,112]. It works closely with the product of the *PARK6* gene—PTEN-induced putative kinase 1 (PINK1)—which depolarizes mitochondria to induce mitophagy. Mutations in both *PINK1* and *Parkin* result in mitochondrial dysfunctions linked to EOPD [113,114,115,116,117].

Together with *PARK1*, *PARK2*, and *PARK6*, other genetic mutations have been detected in PD patients within the so-called “PARK” genes.

DJ-1, encoded by the *PARK7* gene, is a ubiquitous protein principally expressed by neural and glial cells, whose function is not well understood. It is mainly involved in the protection against oxidative stress, as mutations in this gene might cause mitochondrial dysfunctions [118,119,120,121,122].

The Leucine-rich repeat kinase 2 (LRRK2), encoded by *PARK8* gene, when mutated, acquires a gain of function of its catalytic activity, due to cis-phosphorylation (or autophosphorylation) and trans-phosphorylation. The point mutation G2019S is one of the most common LRRK2 mutations found in the genetic forms of PD. However, not all people carrying this mutation have the same risk to develop PD, and different strategies are currently evaluated to find a method to better predict the risk of PD when expressing the G2019S-LRRK2 mutant [123].

Glucocerebrosidase (GCase) is a lysosomal glycoside enzyme encoded by the *GBA1* gene and, although it does not belong to the “PARK” genes, it represents a common genetic risk factor for PD. GCase catalyzes the hydrolytic cleavage of the β-glycosidic bond of glucosylceramide (GlcCer), producing free ceramide and glucose [124]. Thus, GCase plays a central role in the degradation of complex lipids and the overall turnover of cellular membranes. Deficiencies in this enzyme lead to accumulation of GlcCer and to the development of the lysosomal storage disease, known as Gaucher′s disease [125]. It has been speculated that alterations in GCase functions lead to a decreased lysosomal/proteolytic activity resulting in a structural change of α-Syn, from soluble to aggregated [126]. In the contest of PD, *GBA1* mutations are associated with the appearance of cognitive impairments and motor disabilities [127,128,129]. Other genes have been discovered to play a role in the pathogenesis of PD, including *UCHL1* (*PARK5*) [130] and *ATP13A2* (*PARK9*) [95], as reviewed elsewhere [94].

### 2.2. Gut Microbiota and PD

More recently, other factors have been correlated with PD, including the specific gastrointestinal microbiota composition. Gut microbiota has been reported to have a pivotal role in the development of several pathologies, including cancer, inflammation and NDs [131,132]. In particular, the gastrointestinal microbial population may play a crucial role in influencing the brain via a two-way interaction with the neural, neuroendocrine and immune systems, in a network called “gut-brain” axis [133]. Several studies highlighted significant differences existing between the intestinal microbial population in PD patients vs. healthy controls. These alterations mainly involve the diversity and the abundance of some species, such as Lactobacilli, Fecalibacteria and others, which can be over- or under-represented in PD-affected patients [134,135,136,137]. Additionally, the commonly stomach-infecting pathogen *Helicobacter pylori*, causative agent of peptic ulcers, has been recently associated with PD. It has been demonstrated that these bacteria may affect the absorption of levodopa (l-Dopa), thus causing motor fluctuations in PD patients [138,139,140]. In general, imbalances in the intestinal microflora—condition called dysbiosis—and, in particular, a small intestinal bacterial overgrowth may be correlated with gastrointestinal symptoms and motor function impairment in PD-affected subjects [141,142]. Moreover, the alteration of the intestinal permeability and the impairment of the natural intestinal barrier could facilitate the action of toxins deriving from inner or outer intestinal environment, resulting in an enhanced inflammatory response within the Enteric Nervous System (ENS) [143]. This finally may trigger an exacerbated immune response both at the intestinal and at the CNS levels [144].

Importantly, intestinal dysfunction (i.e., constipation) is one of the most common non-motor symptoms of PD, even years before CNS degeneration, thus representing an early indicator of PD onset [145]. In 2012, Shannon and colleagues discovered that α-Syn aggregates were present in the colon tissue before the onset of PD [145]. This observation led to the theory that PD could originate within the ENS, and hence spread to the vague nerve, reaching the CNS [146]. Additional in vivo studies gave further strength to the theory of an intestinal origin for PD [143,147,148]. Even an abnormal pro-inflammatory activity, caused by the inflammatory bowel disease (IBD), may lead to hyperinflammation, dysfunctional immune response and, finally, favor the onset of PD [149,150,151,152]. For all these reasons, different strategies to manipulate the gut microbiota composition, including the use of probiotics, are currently under study as valuable adjuvants in the treatment of PD (see below). This approach may contribute to decrease the local gastrointestinal inflammation and subsequent ROS-related impairment, as demonstrated by several clinical observations [153].

### 2.3. PD Signature Markers in EVs

In 2006, a study from Théry and colleagues demonstrated for the first time the presence of EVs in biological fluids, such as blood and urine [154]. Since then, several protocols have been developed to isolate and purify biofluid-derived EVs—which stably protect their cargoes from degradation—for the discovery of novel vesicle-associated biomarkers [155]. Virtually all biofluids are considered important sources of vesicles, where also CNS-derived EVs can be detected.

So far, in PD studies, EVs have been isolated from cerebrospinal fluid (CSF), plasma, serum, saliva, and urine. In addition, EVs have been isolated, in vitro, from the culture supernatant deriving from a variety of cell types, including brain and peripheral cells [55]. As such, EVs and EV-derived cargoes may be relevant as diagnostic and/or prognostic tools in PD, as herein summarized.

Many of the above-mentioned defective/mutated PD proteins have been seen associated with circulating EVs. The possibility to noninvasively evaluate the presence of such molecules captured the interest of researchers who actively investigate EV-associated PD biomarkers [156]. The first report indicating that α-Syn is secreted inside EVs dates back to 2005 [157]. The presence of α-Syn in the vesicular fraction may have two effects in the context of PD: (i) to prevent the accumulation of aggregates inside the cell [158]; but also (ii) to spread the pathology to other districts [159,160]. EV-associated α-Syn has been detected in saliva, plasma, CSF, and serum from PD patients. Although sometimes its levels were found controversial in different reports, it is possible to envisage the use of EV-α-Syn as a valid PD biomarker in the near future [161,162,163,164,165,166,167,168,169,170,171].

In addition to α-Syn, LRRK2 has been discovered inside EVs, especially the mutated form G2019S which displays phosphorylation at the Ser(P)-1292 residue. The functions associated with this protein are still not well defined, although it seems to be involved in the regulation of biogenesis of vesicular and membranous cellular structures [172,173]. The levels of Ser(P)-1292-LRRK2 has been found higher in EVs from urine samples of PD patients compared to healthy controls, and reflected the severity of the disease [174]. Also, the presence of DJ-1 inside EVs has been analyzed in urine and plasma samples from PD patients. EV-DJ-1 levels are higher in PD subjects compared to controls, even if further studies need to confirm its validity as a novel PD biomarker [164,175,176]. Another interesting finding about EV-based biomarkers can be ascribed to the presence of cellular prion protein (PrPC) in EVs derived from PD patient plasma samples [177]. PrPC is a glycosylphosphatidylinositol-anchored membrane protein mainly located in the CNS and involved in the transmission of α-Syn to neurons [178]. Recently, in 2020, Leng and colleagues demonstrated that the levels of EVs-PrPC were higher in PD patients compared to controls and correlated with the progression of the cognitive decline [177].

Besides dysfunctional proteins, specific small non-coding RNAs (i.e., miRNAs) have been associated with the development and/or the progression of PD. Of note, the profiling of EV-miRNAs in both serum and CSF bear the potential to become a reliable diagnostic tool for PD [179,180].

Finally, very recent reports showed that gut microbiota-derived EVs may have specific effects in several pathologies, including NDs. More specifically, bacteria-derived EVs in blood have been found dramatically altered in an AD mouse model, supporting their use for the metagenomic analysis of the gut microbiota in AD [181]. Similar findings were obtained using mucosal-luminal interface samples from pediatric IBD patients, suggesting that the alteration of intestinal microbe-derived EVs may be associated with an aberrant host-microbiota interaction also in other inflammatory conditions, including PD [182].

It is evident that a better understanding of the different risk factors in PD—and their relative contribution to the onset and progression of the disease—is urgently needed. The precise identification of the molecular triggers responsible for the DAergic neurodegeneration will help to predict, and possibly to prevent, PD. This is a key point, as PD is usually diagnosed when already 70% of the neurons in the SNpc are lost, making their recovery a major challenge for regenerative medicine.

In this scenario, the identification of novel biomarkers associated with EVs may help the early diagnosis of PD. Importantly, compared with biomarkers identified in conventional specimens, EV-associated biomarkers may provide the highest amount of sensitivity and specificity, which can be attributed to their excellent stability in biofluids.

## 3. Therapeutic Approaches for the Treatment of PD

At present, DAergic drugs designed to replace the action of DA in the depleted striatum represent the pharmacological treatment of PD [183,184,185,186]. Currently, various options are available to achieve this goal, either indirectly—through drugs acting at a presynaptic level, via DA metabolism, inhibiting the breakdown of endogenous DA—or directly, through DAergic agonists acting at a post-synaptic DAergic receptor level [187,188,189,190,191,192]. Unfortunately, there are no effective neuroprotective therapies for PD, but different clinical trials designed to develop potential disease-modifying strategies are currently on their way [12,95]. The complex topic of pharmacological therapies and PD management is out of the scope of this work and recent reviews have expanded on the pharmacological advances in PD therapeutics, including medication regimens tailored to the individual patient, based on the severity and temporal nature of their symptoms, as well as the side effects that they experience and how to manage them [12,95,183,184,185,186,187,188,189,190,191,192,193,194]. Numerous other medications have a role as adjunctive treatment, including surgical treatments, such as deep brain stimulation (DBS). These approaches are being assessed both to provide relief to patients with advanced PD, as well as to ameliorate their quality of life [194]. Below, the advances in cell-based therapies will be discussed, with major emphasis on EV-related approaches, either by targeting key PD pathogenic mechanisms or/and by slowing or preventing its progression.

### Cell Therapies

Cell therapy—consisting of the transplantation of relevant cell types—was proposed following the establishment of the 6-OHDA-animal disease model in the 1970s [195]. 6-OHDA is a synthetic neurotoxic compound widely used to reproduce the nigrostriatal lesion characteristic of PD. Since this molecule cannot pass the blood–brain barrier (BBB), it is intracranially injected and, through the DA active transporter (DAT), it induces neuronal death within the ventral midbrain with consequent DA reduction and insurgence of motor dysfunctions [196]. The selective and irreversible lesion of the nigrostriatal pathway produced by 6-OHDA may be used as a useful parameter to evaluate the restoration of the DAergic tone following cell transplantation [197].

The first clinical trials evaluating cell therapy in PD were conducted in the 1980s, by using DA-producing adrenergic medullary cells. The results were not satisfactory, as the cells poorly grafted in the caudate nucleus and patients experienced postoperative psychiatric disturbances [197]. More interesting results were instead achieved by transplanting human fetal ventral mesencephalic tissue into the striatum of PD patients. Although some patients displayed a long-term efficacy, most of them developed graft-induced dyskinesia (GID) that, together with the ethical and safety issues connected to the use of fetal tissue, made this approach not feasible for many years [197]. Nevertheless, in 2019, this particular branch of cell therapy was reconsidered by the TRANSEURO consortium, which developed a currently ongoing open-label study (NCT01898390), in which 11 patients with mild PD will be transplanted with human fetal DAergic cells [198].

However, in the context of cell therapy, the use of stem cells represents the most promising approach for transplantation in PD, as they may be differentiated into specialized phenotypes, such as DAergic neurons. Moreover, these cells, thanks to their neurotrophic/immunomodulatory properties, are also able to reduce inflammation and inhibit apoptosis of damaged tissues.

Embryonic stem cells (ESCs) were the first type evaluated for transplantation in 1998 [199]. Again, the adverse immune reactions and ethical issues limited their use [200], although an ongoing phase I/IIa clinical trial is evaluating the safety and the efficacy of intracranial transplantation of human ESC-derived neural precursor cells in PD patients (NCT03119636), following their positive outcome in primate PD models [201].

Induced pluripotent stem cells (iPSCs) represent a valid alternative for cell therapy in PD. These cells are collected from skin (fibroblasts) or red blood cells and they are reprogrammed to differentiate into DAergic neurons or replicative astrocytes [200,202]. This can be achieved by transfection with lentiviral vectors carrying specific transcription factors [203], or by exposure to specific growth conditions [200,202]. Although these cells are recovered from the same patients, thus overcoming the immune rejection problems, teratoma formations have been observed after transplantation, leaving open questions about their safety [204,205]. On the contrary, other reports demonstrated that iPSC-derived DAergic progenitor cells transplanted in primate PD models survived and worked as DAergic neurons without any tumor formation in the brain, therefore demonstrating their clinical relevance for transplantation in PD patients [206,207].

Another strategy to achieve the rescue of damaged DAergic neurons was evaluated in pre-clinical models by transplanting adult syngeneic neural progenitor stem cells (NSCs) in the SNpc of aged MPTP-treated mice [55]. One third of these cells acquired an astrocytic phenotype and, synergistically with endogenous astrocytes, they activated the Wnt/β-catenin signaling in SNpc-DAergic neurons, finally inducing neurorescue and immunomodulation [55]. Remarkably, a robust migration of NSCs and NSC-derived astrocytes to the Wnt-sensitive midbrain DAergic niche was accompanied by a time-dependent DAergic neurorescue [55]. Importantly, in NSC-grafted mice, the NSC-derived astrocytes and the endogenous astrocytes expressed Wnt1, mediating DAergic neurorescue and microglia down-regulation [55]. Following a similar line of research, Serapide and coworkers addressed the ability of ventral midbrain astrocytes, used as a graft source for unilateral transplantation above the SN of middle-aged MPTP mice, to ameliorate the aged and MPTP-injured microenvironment, thus mitigating nigrostriatal toxicity [23]. Here, grafting VMB astrocytes rejuvenated the SN microenvironment via a downmodulation of microglial pro-inflammatory status. These data suggest a chief role for VMB astrocytes and astrocyte-derived molecules in favoring neurorepair in PD [23].

A first phase I clinical study, which evaluated the safety and the efficacy of human parthenogenetic derived NSCs (ISC-hpNSC) as therapy for PD, started in 2015 in Australia. These cells were intracerebrally implanted into the striatum and SNpc of individuals affected by moderate to severe PD (NCT02452723). This intrinsic ability of NSCs to induce neurorepair and immunomodulation may be ascribed, at least in part, to NSC-derived EVs that could be used as substitute in a cell-free approach (see below) [208,209]. In line with these findings, another ongoing clinical trial is evaluating the safety and the efficacy of human fetal NSCs injected intranasally in PD patients (NCT03128450).

Mesenchymal stem cells (MSCs) are another cell type that potentially might be used for PD cell therapy. These cells have a great plasticity and easily integrate into the host tissue as demonstrated in different contexts, including AD [210], ALS [211], multiple sclerosis (MS) [212], autoimmune diseases [213], diabetes [214], and PD [215]. Several studies demonstrated their potential for trans-differentiation in DAergic neuronal phenotypes [216], and two ongoing clinical trials are evaluating the efficacy and the safety of undifferentiated or differentiated umbilical cord-derived MSCs in PD patients. These cells are being administered intravenously (NCT03550183) or intrathecally following their differentiation in NSCs (NCT03684122). As already mentioned for NSCs, the benefits of MSCs seem to be mediated also via their secretome, including the EV component, as demonstrated by several studies [217,218,219]. Despite MSCs transplantation ameliorated PD symptoms, the trophic effects were often only transient [220,221]. Moreover, in some cases, the systemic injection of these cells showed severe side effects, such as pulmonary thrombosis [222,223,224,225], whereas the alternative intracranial transplantation is a very invasive procedure [215].

Overall, the issues and risks potentially connected with the implantation of “living materials” within the CNS stimulated the scientists to evaluate alternative strategies for brain repair. Indeed, different cell-free strategies are under study to ameliorate the clinical picture in PD patients. For instance, lentivirus-based gene therapies have been evaluated in phase I–II PD clinical trials with ProSavin [226], a lentiviral vector carrying three enzymes for DA biosynthesis [227]. The safety of this approach was demonstrated together with motor improvements in mid- to late-stage PD patients. However, DA replacement needs to be better addressed to maximize the benefits and, again, intracranial injection is a highly invasive procedure [228].

## 4. EV-Based Therapeutics for Cell-Free Treatment of PD

Following the first pioneer studies, the subsequent functional characterization of the EVs remained elusive for many years, with scarce interest in the field. In 1996, an important publication from Raposo and colleagues, reported for the first time the presence of surface antigens on EVs released by B lymphocytes. Importantly, the EVs were able to induce a specific T-cell response [229]. On the same line, Zitvogel et al. in 1998 demonstrated that dendritic cells (DCs) release antigen-presenting vesicles, carrying major histocompatibility complex proteins and T-cell co-stimulating cytokines and chemokines [230].

The EV field has grown exponentially after these seminal findings, and several clinical trials are currently evaluating the efficacy of the EV-based treatments for different pathologies [231], including the recent Severe Acute Respiratory Syndrome Coronavirus 2 (SARS-CoV-2) infection (NCT04276987).

In the context of CNS, one of the main challenges for candidate PD drugs is to cross the BBB and to target the brain. This is potentially achievable by using lipid-based vehicles, such as EVs, which display an innate low immunogenicity and an intrinsic ability to cross biological barriers [232]. Moreover, an increasing body of evidence supports the neuroprotective/neuroregenerative potential of EVs, with specific therapeutically relevant outcomes depending on the donor cells and the microenvironmental milieu [50,51].

**Figure 2:**
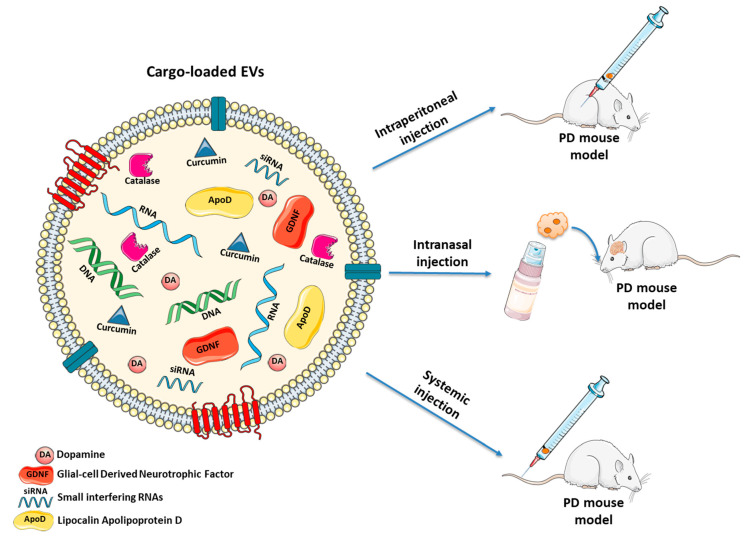
Naturally occurring and engineered EVs as cell-free treatment for PD. EVs may be manipulated to deliver: (i) anti-oxidant agents (e.g., curcumin, catalase or ApoD) which protect neurons from oxidative stress; (ii) growth factors (e.g., GDNF) to stimulate proliferation of DAergic neurons; (iii) DA to ameliorate behavioral parameters; (iv) siRNAs silencing the expression of SNCA gene to decrease α-Syn levels. Different routes of administration (systemic injection, intranasal injection and intraperitoneal injection) may be used to treat PD mouse models.

During the last decade, several strategies based on natural or modified EVs have been proposed, also for treating PD [233] (Figure 2 and Table 1).

### 4.1. Non-Modified EVs to Arrest the Pathologic Propagation of PD

Several studies showed the promising effects of cell therapy for PD treatment. In line with these encouraging results, EVs derived from clinically relevant cells have been explored to reproduce the beneficial effects mediated by the corresponding donor cells, thereby reducing the possible side effects linked with the administration of whole cells. For example, a particular kind of MSCs—recovered from the dental pulp of human exfoliated deciduous teeth (SHEDs)—has been used by Jarmalavičiūtė and colleagues in 2015 [234]. These particular cells, arising from the embryo neural crest, have unique neurogenic properties, as they are able to differentiate into DAergic neuron-like cells and Schwann cells [235]. Based on these properties, the authors selected SHEDs as EV donor cells. The ReNCell VM immortalized human neural progenitor cell line—opportunely differentiated to assume a DAergic neuronal phenotype—was chosen as model of target cell, for evaluating the potential neuroprotective effects of SHED-derived EVs. To reproduce the parkinsonian/oxidative environment, the neurons were treated with the neurotoxin 6-OHDA [234]. The authors compared two strategies to culture SHEDs: (i) classic growth on two-dimensional flasks in serum free medium; and (ii) three-dimension growth on laminin-coated micro-carriers into a bioreactor, in a suspension-like environment. They also analyzed vesicles obtained with two different protocols: 20,000× *g* centrifugation (l-EVs) and 100,000× *g* centrifugation (s-EVs). Interestingly, the authors demonstrated that s-EVs only, specifically derived from SHEDs cultured on 3D micro-carriers, were able to suppress apoptosis of 6-OHDA treated neurons. In fact, this anti-apoptotic effect was not observed with s-EVs deriving from SHEDs cultured with standard methods, demonstrating that the culture conditions may affect the nature and amount of cargoes contained inside the EVs [234,251].

Recently, in 2019, the same group published a follow-up work where they evaluated the effect of SHED-EVs in vivo, in a PD rat model [235]. The animals were subjected to unilateral cranial injection of 6-OHDA in the medial forebrain bundle and then intranasally treated with EVs. By using the apomorphine test, the authors evaluated the effects of SHED-EVs on the dysfunctional consequences deriving from 6-OHDA mediated striatal lesions. Apomorphine injection is known to induce involuntary contralateral rotations in 6-OHDA lesioned animals, while controls do not rotate at all. Importantly, the treatment with SHED-EVs in 6-OHDA treated rats halved the number of contralateral rotations in the apomorphine test, together with a significant improvement in gait parameters. Moreover, a notable increase in the density of tyrosine hydroxylase (TH, the rate-limiting enzyme converting l-tyrosine to l-Dopa) positive cells was observed in both the striatum and the SNpc of 6-OHDA rats, as a result of the EV treatment [235].

The authors suggested that the neuroprotective effects of SHED-EVs might primarily due to their protein content: for instance anti-oxidant molecules—such as the superoxide dismutase 1 (SOD1) [252], the thioredoxin (TXN), the peroxiredoxin-6 (PRDX6) and HSP70—but also other proteins belonging to the family of annexins. Thanks to the above-listed features, EVs have been proposed by the authors as potent tools for a safe and non-invasive therapy to delay the progression of the pathology and to improve the motor impairments in PD patients [235]. It is important to highlight the potential combinatorial effects mediated by the diversity of EV-cargoes, which represents one of the most relevant advantage for EV-based therapeutics, as reported in several contexts [253].

Remarkably, besides SHED-EVs, glial-derived EVs may play key roles in both neurodegeneration and neuroprotection in PD experimental models, as recently reviewed by our group [51]. For example, the studies of Leggio and collaborators [254] revealed that astrocytes from the ventral midbrain, which induce nigrostriatal rescue in MPTP-treated mice [23], were able to secrete vesicles in the size range of s-EVs and positive for classical EV markers (e.g., CD63, CD9, Tsg101) [254]. Interestingly, in basal conditions, ventral midbrain astrocytes release more EVs than striatal astrocytes. This diversity in the secretion of EVs seems to suggest the existence of specific brain region-linked differences which may have possible functional implications in the intercellular communication within the nigrostriatal area [254]. Moreover, the authors identified a specific subset of miRNAs and proteins enriched in the ventral midbrain astrocyte-derived EVs, with a potential impact on DAergic neuroprotection and NSC differentiation.

Overall, these data support the importance to further characterize the potential of astrocyte-derived EVs as advanced therapeutics tools for promoting neuroprotection and neurorepair in PD (Leggio et al., manuscript in preparation).

### 4.2. EVs and Lipophilic Particles as Drug Delivery System in PD

#### 4.2.1. DA Replacement

As mentioned above, DA cannot be administered directly to PD patients given its inability to cross the BBB. Other prodrugs, including l-Dopa, have been developed to address this issue. Moreover, novel approaches based on lipophilic coating have been further investigated. In 2015, Pahuja and colleagues prepared nanoparticles (NPs) of poly(lactic-coglycolicacid) (PLGA) incorporating DA. This material has been chosen given the high biocompatibility. In fact, its degradation products (i.e., lactic and glycolic acids) are extremely biodegradable since they are eliminated as carbon dioxide and water through the Krebs cycle. DA, once incorporated within the NPs, was more stable in the blood and with an improved capability of entering the brain. Importantly, DA-NPs, when injected in the 6-OHDA PD rat model, reversed neurobehavioral and neurochemical impairments of injured animals without any increase in ROS production. No additional neurodegeneration in the striatum and in the SNpc was observed, thus suggesting DA-NPs as a viable approach for a sustained delivery of DA in the brain [236].

A similar technology has been characterized by Qu and colleagues in 2018 [237]. They prepared fresh serum-derived EVs, which were loaded with DA and injected intravenously into a 6-OHDA mouse model. To evaluate the brain-targeting potential, EVs were labeled with the lipophilic fluorescent compound 1,1′-Dioctadecyl-3,3,3′,3′-Tetramethylindodicarbocyanine, 4-Chlorobenzenesulfonate Salt (DiD). As expected, 6 h post-injection, EVs were detected in both the striatum and SNpc. The therapeutic efficacy was instead evaluated three weeks following DA-EV administration. The results showed a significant amelioration of behavioral parameters compared to 6-OHDA control mice. Importantly, an enhanced accumulation of DA was observed specifically in the striatum. Moreover, the number of TH-positive cells in the lesioned striatum raised, demonstrating that this approach was efficient and safe [237].

One year later, Tang and colleagues synthetized borneol and lactoferrin-modified nanoparticles (Lf-BNPs) used as DA carrier. The authors intranasally administered the DA-Lf-BNPs to 6-OHDA PD rats, showing that these NPs restored the DA levels in the striatum of PD animals. As a consequence, a decrease in the number of contralateral rotations via the apomorphine test was also observed in treated rats, demonstrating the efficacy of nanoparticle-mediated DA delivery [238].

#### 4.2.2. Anti-Oxidant Agents

Considering the pivotal role played by oxidative stress in the progression of PD, many studies are currently evaluating feasible strategies to inhibit the exacerbated ROS production to restore their balance in the brain. For this purpose, recent in vitro and in vivo studies have been carried out to find novel approaches to scavenge ROS.

Curcumin is a natural polyphenol widely used as anti-oxidant, antiseptic, antibacterial and cytostatic agent in several diseases [255]. Curcumin can also protect neurons from oxidative stress, by inhibiting protein aggregation and promoting neurogenesis in vivo [256]. However, its poor solubility significantly reduces the overall bioavailability. Taking advantage from its hydrophobicity and the preferential interaction with lipid membranes, the group of Zhang in 2010 and 2011 developed an EV-based model to encapsulate curcumin [239,240]. First, they assessed the increased solubility and stability of curcumin-EVs in vitro and in vivo [239]. Then, to evaluate the anti-inflammatory properties of curcumin encapsulated within EVs, they delivered intranasally curcumin-EVs in mice challenged with LPS, used as a model of neuroinflammation. The authors observed a reduction of microgliosis following the treatment, thereby supporting the possibility to use curcumin-EVs to treat NDs [240]. Notably, the promising results obtained by using curcumin-loaded vesicles have been translated into clinics with an ongoing phase I clinical trial in cancer patients (NCT01294072). In the study, curcumin-EVs are orally administered, as diet supplement, to patients diagnosed with colon cancer [257].

Another molecule with important anti-oxidant activity is the enzyme catalase, which hydrolyzes the hydrogen peroxide to form hydrogen and oxygen [258]. For this purpose, Haney and colleagues in 2013 developed a cell-based delivery system in order to induce the catalase expression in brain cells [241]. They used the macrophage cell line Raw 264.7 stably transfected with a plasmid encoding a fluorescent catalase. These cells were intravenously injected into the 6-OHDA PD mice. Once in the brain, the cells were able to induce profound anti-inflammatory and neuroprotective effects, as confirmed by the improved motor functions [241]. To evaluate whether the neuroprotective effects were mediated by macrophage-derived EVs, the authors isolated and treated the PD animals with the vesicles produced by macrophages overexpressing the fluorescent catalase. EVs were shown to carry the catalase at DNA, RNA, and protein levels. This important finding supports the notion that cells can exchange both nucleic acids and proteins by using their EVs as carriers. The authors demonstrated that contiguous neurons can efficiently transfer EVs, resulting in de novo protein synthesis in target cells, in turn responsible to counteract the inflammation [241].

Two years later, in 2015, the same group used again the Raw 264.7 macrophages to obtain EVs loaded with catalase by using saponin-based permeabilization. These vesicles were intranasally injected into 6-OHDA-treated mice and the effects were evaluated 21 days later. First, they found reduced microgliosis and astrocytosis in mice treated with catalase-loaded EVs compared to free catalase treated mice. In addition, the authors observed a significant increase of DAergic neurons in EV-treated mice. Moreover, in the apomorphine test, PD mice intranasally injected with catalase-loaded EVs rotated less than control mice. These results demonstrated that the EVs improved the bioavailability, as well as the targeting capability of catalase in the inflamed brain, with protective effects towards DAergic neurons [242].

In 2018, Kojima et al., developed an interesting cell-based system equipped with an “EV production booster” [243]. They used the HEK293T cell line as donor cells to produce EVs loaded with the catalase mRNA, to specifically target the brain. As expected, these vesicles attenuated both the neuroinflammation and the neuronal death induced by the 6-OHDA treatment [243].

More recently, in 2019, Pascua-Maestro and colleagues focused on another relevant player in the context of neuroinflammation, evaluating how EVs are involved in the transport of lipocalin apolipoprotein-D (ApoD) from astrocytes to neurons [244]. The role of ApoD in context of CNS has been widely documented. This molecule binds lipids (e.g., cholesterol, arachidonic acid and steroids) and it is highly expressed by glial cells in response to oxidative stress conditions, including PD [259]. ApoD is a small glycoprotein with a strong anti-oxidant activity, since it prevents lipid peroxidation, and therefore protects membranes from the oxidative damage [260]. It was observed that ApoD expression increases in order to respond to the enhanced demand of lipids during brain development [260]. In fact, ApoD expression is five to ten-fold higher in adult brains compared to neonatal ones, following expression changes which occurs during brain maturation and aging [260,261]. ApoD is located inside the endo-lysosomal compartment and, not surprisingly, it is found associated with EVs [244]. In their work, Pascua-Maestro et al. demonstrated that ApoD is conveyed from astrocytes towards neurons carried within the secreted EVs. The authors treated neurons with the herbicide Paraquat, which is a well-known ROS generator (especially the superoxide free radical). Hence, they found that astrocyte-derived EVs were able to restore the viability in damaged neurons, via the direct transfer of ApoD [244]. Strikingly, ApoD was detected in blood-circulating EVs from people affected by inflammatory-related conditions, such as PD patients, further suggesting a potential use of EVs as biomarkers [259].

#### 4.2.3. Inhibition of α-Syn Synthesis/Aggregation

As discussed above, the α-Syn aggregates contribute both to motor and non-motor dysfunctions in PD patients. For this reason, research is trying to mitigate the formation and the accumulation of α-Syn in the brain.

Among the methodologies investigated, one of the most explored is the use of microRNAs (miRNAs) or small interfering RNAs (siRNAs), which operate at post-transcriptional level by silencing the expression of the *SNCA* mRNA [180,262,263,264]. The first report describing the EV-mediated delivery of siRNAs to specific target sites have been proposed by Alvarez-Erviti and colleagues in 2011 [265]. To avoid the development of adverse immune reactions in the host, they used immature dendritic cells to produce EVs. Notably, the authors developed a molecular system to increase the EV targeting to the brain. For this purpose, they fused the rabies viral glycoprotein peptide (RVG)—which specifically binds the acetylcholine receptor within the CNS—to the *N*-term of the exosomal protein Lamp2. This construct was used to transfect the dendritic cells before EV isolation. Once obtained the RVG/Lamp2-modified vesicles, the authors used the electroporation to introduce siRNAs within EVs [265].

The same protocol was used by the same group in 2014, to deliver α-Syn siRNAs to the brain of transgenic mice expressing the human phospho-mimic S129D α-Syn. When systematically injected in normal mice, EVs loaded with α-Syn siRNAs were able to reach the midbrain, the striatum, and other cortical brain areas, where the siRNAs produced a significant decrease of α-Syn at both mRNA and protein levels. Importantly, when injected in transgenic mice, these vesicles induced a complete depletion of α-Syn mRNA, but they were less effective at the protein level. The human α-Syn S129D protein is more prone to aggregate, and probably the presence of α-Syn inclusions requires a prolonged down-regulation of the expression to obtain an efficient depletion. Moreover, as described by the authors, this siRNA-based treatment demonstrated a short-term efficacy [245].

Five years later, in 2019, the same group designed a novel short hairpin RNA (shRNA) minicircle (MCs) specifically delivered in the brain through RVG modified EVs [246]. MCs are small double-stranded DNA vectors that can be easily inserted inside the EVs. In fact, MCs contain only the transgene cassette, favoring a higher expression of the transgene and thus allowing a prolonged down-regulation of the target gene [266,267]. The authors used again the transgenic mouse models expressing the human phospho-mimic S129D α-Syn to evaluate the efficacy of shRNA-MCs-loaded EVs injected intravenously. This time they obtained a prolonged down-regulation of S129D α-Syn mRNA and a significantly lower level of α-Syn protein, compared to controls. Notably, these brain-targeting EVs were able to down-regulate the α-Syn expression in several areas of the CNS, where they prevented protein aggregation and consequently the death of neurons [246]. Moreover, the authors tried to evaluate the effect of this approach on PD mice subjected to intrastriatal injections of murine α-Syn pre-formed fibrils. Even in this case, the α-Syn expression was efficiently down-regulated, at both mRNA and protein level compared to controls, with a substantial protection against DAergic terminal loss in the striatum and a consequent improvement of the clinical parameters [246]. Taken together, these data supported the great potential of this long-term therapy based on EV systemic administration. However, as discussed by the authors, this approach is effective at the early stages of the pathology, and therefore the efficacy during PD progression remains to be further evaluated [246].

More recently, in 2020, Zhao et al. developed a nanoparticle (NP)-based system able to inhibit α-Syn aggregation and also to attenuate microgliosis [247]. In fact, as mentioned earlier, upon sensing oxidative stress, microglia become activated, which may further exacerbate neuroinflammation. In addition, microglia play a crucial role in α-Syn clearance and degradation. NP preparation included two anti-oxidant molecules, the ferulic acid diacid (FAA, a strong anti-inflammatory molecule), and the tannic acid (TA, a protein aggregation inhibitor). Following in vitro studies, the authors observed that these NPs were able to inhibit fibrillation and aggregation of acetylated α-Syn (Ac-α-Syn, one of the most common post-translational modification of α-Syn, with a potentially relevant pathological role). Moreover, the authors applied these NPs on BV2 microglial cells previously treated with monomers of Ac-α-Syn or mutated A53T α-Syn. The results showed that in the presence of NPs, the levels of intracellular oligomer formation significantly decreased for both Ac-α-Syn and A53T α-Syn triggers. To evaluate microgliosis attenuation, BV2 cells were stimulated with A53T α-Syn, which increased the production of pro-inflammatory molecules, such as ROS, TNF-α and IL-6. NP treatment lowered the secretion of TNF-α and IL-6, as well as ROS production, thus confirming the attenuation of α-Syn induced microgliosis in the presence of FAA-TA NPs [247]. Considering that few approaches have been designed to target both α-Syn aggregation and microglial activation, this work represents a valid anti-oxidant-based nanotherapeutic alternative to treat NDs, such as PD.

### 4.3. GDNF Therapy

As illustrated above, GDNF is one of the “beneficial” growth factors produced by astroglial cells that stimulates proliferation and provides support and protection to DAergic neurons. Following the activation of astrocytes and microglia during neurodegeneration, GDNF levels drop. In vitro and in vivo studies aiming to restore GDNF levels demonstrated the great potential of this approach. Consequently, GDNF administration to PD patients led to promising results [268,269]. Moreover, the beneficial effects of GDNF on neuronal survival has been recently evaluated in 6-OHDA rodent models transplanted with ESC-derived DAergic neurons [270,271]. However, the invasiveness of the procedure (i.e., the intracranial infusion) makes this approach not ideal for its usage in clinical routine [272]. For this reason, other strategies have been proposed to reduce the invasiveness and guarantee the safety.

In 2010, an alternative route for GDNF administration has been developed by Biju and colleagues. They transfected ex vivo bone marrow stem cell-derived macrophages transduced with a lentiviral vector overexpressing GDNF. These cells were then transplanted in mice before the MPTP-mediated DAergic neuron degeneration. The transplanted cells, which were able to cross the BBB, were recruited within SN area lesioned by MPTP and efficiently differentiated in microglial cells. The results showed a remarkable neuroprotection of DAergic neurons together with an increase of DA production, which, in turn, resulted in motor function improvements [248].

Based on these results, the group of Batrakova and colleagues—the same team investigating the role of catalase in macrophage-derived EVs [241]—in 2014, developed an improved system based on autologous macrophages transfected ex vivo to produce GDNF [249]. Moreover, to avoid the triggering of an M1 pro-inflammatory status, macrophages were cultured in the presence of IL-2. The treatment with this cytokine induces the M2 phenotype, as already discussed for the M1/M2 microglia polarization. The deriving M2 GDNF-producing macrophages were intravenously injected in the 6-OHDA PD mouse model, where they were able to cross exclusively the BBB of inflamed brains, inducing both neuroprotective and anti-inflammatory effects with consequent motor function improvements. As in [241], to evaluate the possible role played by macrophage-derived EVs, the authors analyzed the content of vesicles, finding a significant enrichment in GDNF carried by the vesicles. Therefore, they suggested that EVs might be directly involved in the release of GDNF within the proximity of damaged neuronal membranes, thereby favoring GDNF binding to its receptor expressed by the target neuronal cells. As a consequence, GDNF pathway was activated in recipient neurons with subsequent enhancement of the downstream protective effects [249].

Another strategy to increase GDNF levels in the inflamed brain has been developed in 2019 by Aly et al. [250]. They induced brain cell transfection in vivo by delivering plasmid DNA (pDNA) encoding GDNF, either alone or mixed with a gelatine solution to form NPs, in a PD rat model. Both the naked-pDNA and the pDNA-NPs were then intranasally injected in rats one week before the 6-OHDA toxin treatment, and the effects on PD rat brains were analyzed three weeks later. They found that the NPs, but also—to a lesser extent—the naked-pDNA, reached the brain with a consequent significant increase of the GDNF expression. The high concentration of GDNF within the SN protected DAergic neurons and dendritic fibers against the damage induced by 6-OHDA. In addition, the authors observed significant improvements in motor functions [250].

**Figure 3:**
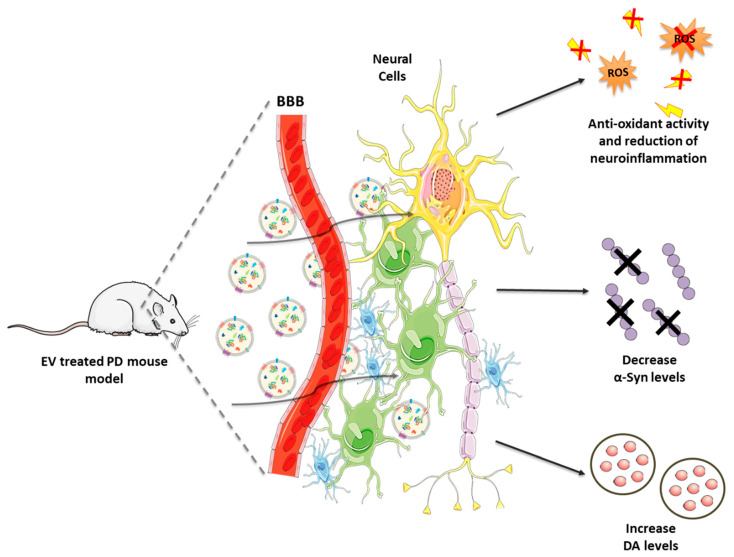
Naturally occurring and engineered EVs in the treatment of PD: mechanism of action. Following their administration, EVs cross the BBB to reach the brain, where they target neurons and glial cells. Among the effects in EV recipient cells: (i) production of anti-oxidant molecules, (ii) reduction of neuroinflammation, (iii) decrease in α-Syn levels, (iv) increase in DA bioavailability.

Therefore, these promising findings corroborate the important role played by GDNF to preserve DAergic neurons from cytotoxic triggers. Moreover, this approach seems to be as effective as invasive intracranial infusion, with the advantage to be more robust and easily applicable. Combining the safety and targeting properties of EVs with the reproducibility of the synthetic NPs could lead to the development of an improved drug delivery system not only for PD, but also for other conditions. Figure 3 illustrates the EV-based therapeutics for PD and the related outcomes are listed in Table 1.

## 5. Conclusions

PD is a progressive and debilitating ND, which mainly affects the elderly people, but it is additionally diagnosed in younger subjects. Despite its long history, the causes of PD remain elusive and no cure is currently available. Moreover, no clear-cut diagnostic biomarkers for PD exist yet, and current methods to track PD progression are missing. Indeed, PD neurodegenerative processes typically begin decades before the appearance of debilitating clinical symptoms. Therefore, the diagnosis is achievable only when most of the relevant neurons have already died. The identification of novel PD biomarkers is key to develop early robust PD diagnostic methodologies, as well as to design neuroprotective therapies for at-risk populations.

In this context, EVs are good candidates as promising biomarkers, as well as functional nanodrug carriers. First, EVs are stably released within the blood and other biofluids, where their cargoes—protected from the action of nucleases and proteases present in the external environment—can be easily isolated. Secondly, thanks to their cargoes, circulating EVs may mirror pathological changes, thus representing potential prognostic tools. This application of EVs is currently tested in clinics, where EVs are explored as “sensors” to follow the outcome of a selected therapeutic treatment [273]. Finally, EVs are currently explored as effective BBB-permeable carriers to deliver in a targeted fashion nanodrugs within PD-damaged brains.

In this scenario, naturally occurring EVs (without any in vitro manipulation) have been demonstrated to bear an intrinsic neuroprotective potential. Of note, EVs derived from relevant cell types, such as astrocytes, were shown to mediate the same (or even improved) therapeutic efficacy, compared with the administration of the corresponding donor cells. Indeed, EVs have a low immunogenicity and they can cross the BBB, thus emerging as novel cell-free nanotherapeutics for brain repair. The beneficial properties displayed by EVs may be conjugated with the possibility to properly engineer them. These vesicles may be opportunely modified to deliver anti-oxidant/inflammatory proteins, as well as DA or molecules able to block α-Syn synthesis or aggregation. Moreover, several routes of administration (e.g., intranasal, intravenous, implantable devices) may improve the delivery and the desired effects. In pre-clinical models of PD, these drug-loaded vesicles can induce anti-inflammatory effects and motor function improvements, ameliorating the overall course of the disease.

The research in the field is moving at a fast pace towards the development of a new generation of vesicles, combining the beneficial properties of EVs with the versatility of artificial NPs, such as liposomes and polymersomes. The availability of several biodegradable materials and the possibility to artificially manipulate their chemical structure (e.g., to improve the drug release efficacy or the targeting efficiency) makes NPs very promising tools for PD treatment. A hybrid natural-artificial vesicle, clearly inspired by naturally occurring vesicles, but enriched with polymers and lipids, may enhance the overall responsiveness of vesicles to environmental changes (e.g., pH, temperature, light) [274,275,276]. Altogether, the development of these new-engineered systems, linked to the increasing knowledge about EV-based theragnostics, bear the potential to fill the gap currently existing in PD diagnosis, staging, and therapy.

## Figures and Tables

**Table 1:** EV-based therapeutics for PD.

Donor Cell/Origin	Vesicle Type	In Vitro Model	Outcomes	Route of Administration	In Vivo Model	Outcomes	REF.
MSCs from the dental pulp of human exfoliated deciduous teeth	EVs	ReNCell VM derived DAergic neurons treated with 6-OHDA	Apoptosis suppression	Intranasal injection	6-OHDA treated rats	Improvements in motor and gait parameters; increase in TH+ neuron density	[234,235]
None	DA-loaded poly (lactic-coglycolicacid) nanoparticles	None		Systemic injection	6-OHDA treated rats	Reversion of neurobehavioral and neurochemical impairments; inhibition of ROS production	[236]
Serum	DA-loaded EVs	None		Systemic injection	6-OHDA treated mice	EVs reached the SNpc and the Striatum; increase in DA accumulation and TH+ neuron density; amelioration of behavioral parameters	[237]
None	DA-loaded borneol and lactoferrin-modified nanoparticle	None		Intranasal injection	6-OHDA treated rats	Restoration of striatal DA levels; motor function improvements	[238]
Mouse lymphoma EL-4 cell line	Curcumin-loaded EVs	LPS treated RAW 264.7 macrophages	Decrease of IL-6 and TNF-α production	Intraperitoneal injection	LPS treated mice	Reduction of CD11b^+^ and Gr-1^+^ cells in lung; lower sera levels of IL-6 and TNF-α	[239]
Mouse lymphoma EL-4 cell line	Curcumin-loaded EVs	None		Intranasal injection	LPS treated mice	Reduction of microgliosis through apoptosis induction	[240]
RAW 264.7 macrophage cell line stably transfected with catalase-carrying plasmid	EVs	None		Systemic injection of cells	6-OHDA treated mice	Anti-inflammatory and neuroprotective effects; improvements in motor functions	[241]
RAW 264.7 macrophage cell line	Catalase-loaded EVs by saponin permeabilization	PC12 neuronal cells treated with 6-OHDA; RAW 264.7 cells treated with LPS and TNF-α	Increase in neuronal viability; decrease in H_2_O_2_ levels in macrophage	Intranasal injection	6-OHDA treated mice	Reduction in microgliosis and astrogliosis; improvement in motor parameters	[242]
HEK293T cell line engineered to produce more EVs	EVs containing catalase mRNA	CHRNA7-positive Neuro2A cells treated with 6-OHDA; neuronal and microglia co-cultures treated with LPS	Partial recovery of 6-OHDA induced neurotoxicity; rescue of neurotoxicity in LPS treated cells	Intracerebral implantation of EV producing cells	6-OHDA treated mice	Reduction of ROS-triggered neuroinflammation and rescue of neuronal death within the striatum where 6-OHDA was injected	[243]
Human astrocytoma 1321N1; primary cortical astrocytes treated with human ApoD		Paraquat treated differentiated SH-SY5Y cells, primary WT or ApoD-KO astrocytes	Increase in neurons and astrocytes viability	None			[244]
Mouse self-dendritic cells transfected with the RVG-Lamp2-flag construct	α-Syn siRNA-loaded EVs	SH-SY5Y expressing human S129D α-Syn-HA	Reduction of α-Syn at protein and mRNA level	Systemic injection	Phosphorylation-mimic S129D α-Syn transgenic mice	Significant short-term decrease in α-Syn mRNA levels in midbrain, striatum, and cortex brain areas	[245]
Mouse self-dendritic cells	Anti-α-Syn ShRNA-MC-loaded EVs	SH-SY5Y expressing mouse α-Syn-HA	Reduction of S129D α-Syn protein	Systemic injection	Phosphorylation-mimic S129D α-Syn transgenic mice; mice injected of α-Syn pre-formed fibrils	Prolonged down-regulation of S129D α-Syn mRNA lower protein levels; down-regulation of α-Syn expression; neuroprotection	[246]
None	Ferulic acid diacid: tannic acid anti-oxidant nanoparticles	BV2 microglial cells treated with Ac-α-Syn or A53T α-Syn and/or pro-inflammatory cytokines	Significant decrease of oligomeric aggregated of Ac-α-Syn and A53T α-Syn oligomers; attenuation of microgliosis	None			[247]
Bone marrow stem cell-derived macrophages ex vivo transfected with GDNF-carrying vector	EVs containing GDNF	None		Systemic injection of cells	MTPT and 6-OHDA treated mice	Neuroprotection of DAergic neurons and increased DA production; motor function improvements	[248,249]
None	Gelatine-based nanoparticles carrying GDNF-pDNA	None		Intranasal injection	6-OHDA treated rats	Transfection of resident brain cells with GDNF increase; protection of DAergic neurons and dendritic fibers in SN; motor function improvements	[250]
